# Service User- and Carer-Reported Measures of Involvement in Mental Health Care Planning: Methodological Quality and Acceptability to Users

**DOI:** 10.3389/fpsyt.2014.00178

**Published:** 2014-12-11

**Authors:** Chris J. Gibbons, Penny E. Bee, Lauren Walker, Owen Price, Karina Lovell

**Affiliations:** ^1^Manchester Centre for Health Psychology, School of Psychological Sciences, University of Manchester, Manchester, UK; ^2^Centre for Primary Care, Institute of Population Health, University of Manchester, Manchester, UK; ^3^School of Nursing Midwifery and Social Work, Institute of Population Health, University of Manchester, Manchester, UK; ^4^NIHR Collaboration for Leadership in Applied Health Research and Care for Greater Manchester (NIHR CLAHRC-GM), University of Manchester, Manchester, UK

**Keywords:** mental health, involvement, carers, patient-centered care, questionnaire, review

## Abstract

**Background:** Increasing service user and carer involvement in mental health care planning is a key healthcare priority but one that is difficult to achieve in practice. To better understand and measure user and carer involvement, it is crucial to have measurement questionnaires that are both psychometrically robust and acceptable to the end user.

**Methods:** We conducted a systematic review using the terms “care plan$,” “mental health,” “user perspective$,” and “user participation” and their linguistic variants as search terms. Databases were searched from inception to November 2012, with an update search at the end of September 2014. We included any articles that described the development, validation or use of a user and/or carer-reported outcome measures of involvement in mental health care planning. We assessed the psychometric quality of each instrument using the “Evaluating the Measurement of Patient-Reported Outcomes” (EMPRO) criteria. Acceptability of each instrument was assessed using novel criteria developed in consultation with a mental health service user and carer consultation group.

**Results:** We identified eleven papers describing the use, development, and/or validation of nine user/carer-reported outcome measures. Psychometric properties were sparsely reported and the questionnaires met few service user/carer-nominated attributes for acceptability. Where reported, basic psychometric statistics were of good quality, indicating that some measures may perform well if subjected to more rigorous psychometric tests. The majority were deemed to be too long for use in practice.

**Discussion:** Multiple instruments are available to measure user/carer involvement in mental health care planning but are either of poor quality or poorly described. Existing measures cannot be considered psychometrically robust by modern standards, and cannot currently be recommended for use. Our review has identified an important knowledge gap, and an urgent need to develop new user and carer measures of care-planning involvement.

## Introduction

Enabling service user and carer involvement in care planning is a principle enshrined by mental health policy (1), yet one that is difficult to fulfill in practice and levels of involvement are rarely reported as adequate. Nationally commissioned surveys report high levels of dissatisfaction with user and carer involvement across inpatient and community settings (2, 3), with qualitative discourse reflecting feelings of user and carer exclusion from care-planning decisions.

Service user involvement in mental health care is a principle that has both philosophical and practice-based drivers. Historically, it has been variously identified as a core component of patient-centered care (4), shared decision-making (5) and patient empowerment (6). More recently, systematic synthesis of multiple small scale studies has suggested that organizational initiatives aimed at enhancing user-involved care planning have the potential to lead to enhanced service development, improved staff attitudes, and increased service user esteem (7). Yet, the apparent lack of policy impact on service quality suggests one of two key things: either that it has been inconsistently translated into practice, and/or that definitions of successful care-planning involvement differ between those delivering and receiving care.

Prior surveys of care-planning quality [such as the CQC (2)] have typically relied on a brief audit of basic objective criteria (such as the presence of a service user’s signature on a care plan) as a proxy indicator of successful user collaboration and involvement. In such service-orientated systems, success is defined primarily in terms of outcome (i.e., the availability of a signed care plan) rather than the quality of the process by which this was achieved. Systematic syntheses (8) suggest that users and carers remain equally if not more sensitive to the relational aspects of care, consistently requesting greater respect, improved information exchange, and more meaningful presence and collaboration in care consultations.

Patient-reported outcome measures (PROMs) provide one route by which to quantify people’s experiences and subjective appraisal of health status and health care. Although there remains some debate over the most appropriate goals for PROMs and the mechanisms by which these might best be achieved, it is generally accepted that such goals should be nominated by the service user, possibly in consultation with informal carers (9). PROMS have a role to play in contemporary health services and service provision. Reviews of the impact of PROMS on routine practice advocate the increased use and development of measures to capture issues of importance to patients and the patient–provider relationship as a potentially effective way of facilitating patient-centered care (10). By definition therefore, their purpose and greatest value can be argued to align closely with the political shift toward collaborative and participatory mental health care.

To be of benefit, such measures need to be developed in a rigorous manner and be both feasible and acceptable to service users and/or research participants. The International Society for Quality of Life Research (ISOQOL) present minimum standards of measurement for patient-reported outcomes which advocate that all outcomes have, as a minimum requirement, justification of their conceptual and measurement models; evidence of reliability and validity (including content, construct and responsiveness); interpretability of scores; translation and demonstrable acceptability that minimizes patient and investigator burden (11). As yet however, standardized population-specific and user-derived criteria for acceptability of mental health PROMS have not been developed.

The aim of the current paper was to systematically identify existing questionnaires for measuring involvement in care planning and mental health services; to assess service user and carer attitudes to these scales and to examine the methodological rigor with which they were developed.

## Materials and Methods

### Systematic review

A systematic review of the literature was conducted to search for existing measures of service user/carer involvement in care planning. Searches were undertaken on multiple electronic health and social science databases: MEDLINE, CINAHL, PsycINFO, EMBASE, HMIC, British Nursing Index, Dissertation Abstracts (accessed via Ovid SP), CENTRAL, CDSR, DARE (accessed via the Cochrane Library), ISI Web of Science including SSCI, and SCIEXPANDED (accessed via Web of Knowledge), ASSIA, IBSS, and Social Services Abstracts (accessed via Proquest).

Search terms were identified via research team discussion, MeSH browsing, scanning of the background literature and consultation with mental health professionals (psychiatrists and mental health nurses) serving on the project advisory’s panel. Search terms were developed for each of the key facets of the research topic, namely care-planning, mental health, user perspectives, and user participation, and subsequently combined with the Boolean operators “OR” as well as “AND” (Box 1). It was acknowledged *a priori* that inconsistent terms may be used to describe patient outcome or service process measures and therefore no restrictions were placed on the search terms in relation to this concept. Searches were limited to articles published in English from database inception to December 2012. An update search was conducted at the end of September 2014. Full copies of the search strategies used in the review are available from the authors.

**Box 1:** **Search Concepts and terms used in the review**.

(1) Mental health	(2) Care planning	(3) Service users
Mental disorder$	Care management	Patient$
Mental difficult$	Case management	User$
Mental health	Care plan$	Consumer$
Mental health service$	Care-planning approach	Client$
Mental illness$	Care program$ approach	Survivor$
Mentally ill	Integrated care pathway$	Carer$
Psychiatric disorder$	Integrated care approach$	
Psychiatric difficult$	Integrated care delivery	
Psychiatric health	Advanced directive$	
Psychiatric illness$	Advance directive$	
Psychiatr$	Treatment plan$	
	Discharge plan$	
	Continuity of care	
	Care continuity	
	Care consistency	
	Care coordination	
	Contingency management	
	Contingency planning	
	Managed care program$	
	Joint crisis plan$	

**(4) Service User Perspectives**	**(5) User-centered processes**	**(6) User- centered outcomes**

Attitude$	Collaborati$	Empowerment
View$	Consultation$	Ownership
Opinion$	Cooperation	Engagement
Belief$	Involvement	Choice
Satisfaction	Partnership	Autonomy
Experience$	Participat$	Trust
	Shared decision making	
	Information sharing	
	Professional-patient relations	
	Physician-patient relations	
	Person-centered	
	Person-centered	
	Person-focused	
	Patient-centered	
	Patient-centered	
	Patient-focused	
	Person-focused	
	Patient-focused	

*$ = Truncation operator that allows a single root to return multiple variants e.g., care plan, care plans, care planning*.

All identified papers were screened by title, abstract, and subject headings against pre-specified eligibility criteria and full text copies of potentially eligible studies obtained. Two reviewers (PB & OP) independently undertook study eligibility judgments, with discrepancies referred to a third member of the project team (KL). Inclusion criteria comprised any report or study focused on the quantification or measurement of service user involvement in care planning in secondary mental healthcare. Care planning was defined as any interaction between a user and health professional for the purposes of discussing or addressing that client’s needs or treatment decisions.

### Stakeholder consultation

All identified and eligible measures were collated and presented at a face to face stakeholder consultation event designed to elicit service user and carer views on their relevance and acceptability. User and carer representatives (*n* = 10) were recruited via adverts placed within NHS services, voluntary organizations, academic courses, and existing contacts of the research team. The group met with the principal aim of developing a set of user-derived criteria against which to rate the acceptability and feasibility of quantitative measures of service user and carer involvement.

Each group member had previously received Masters level training in research methods, and was provided with a copy of the MHRN/NIHR publication “Who decides the definition of a good outcome” (12) This article describes how expert panels made up of people with experience of mental health problems have previously discussed and rated a range of commonly used questionnaires used in mental health research.

Each working group was asked to independently develop a list of concepts or attributes which they felt were important for a user/carer-focused questionnaire measure of involvement. These concepts were subsequently fed back and discussed among the larger consultation group. Priority ratings were allocated to each potential attribute and a single, consensual list of acceptability criteria was generated. Each of the published measures identified in our review was then compared against our newly developed service user/carer-derived criteria to establish its suitability for purpose.

### Psychometric quality

The methodological quality of each measure was separately assessed using the Evaluating the Measurement of Patient-Reported Outcomes (EMPRO) criteria (13, 14). The EMPRO tool is a valid and reliable tool to assess the measurement properties and ease of use of patient-reported outcome (PRO) measures. The tool operationalizes guidelines for measurement quality originally proposed by the Medical Outcomes Trust (MOT) (15) and includes consideration for studies that use item-response theory and related methodologies, such as the Rasch model (16), which is covered in less detail in other criteria [e.g., COSMIN (14)]. Such methodologies are increasingly considered to be superior to “classical test theories” as they can produce linear, unidimensional questionnaire measures (17), have a greater number of “tools” to identify irrelevant or misfitting items (18) and can produce ‘conversation rates’ to transform raw questionnaire scores into interval measures suitable for use in parametric statistics (19, 20).

The full EMPRO criteria were used to assess the included questionnaires. To simplify comparison and provide a “quick-look” figure, each of the 39 categories of the EMPRO measure were given an ordinal scoring system of 0–2 covering the possible categories of “Consideration not met by the questionnaire” (0), “Unclear or partially met” (1), and “Consideration fully met and clearly reported” (2).

## Results

### Synthesizing the primary evidence

**Figure 1:**
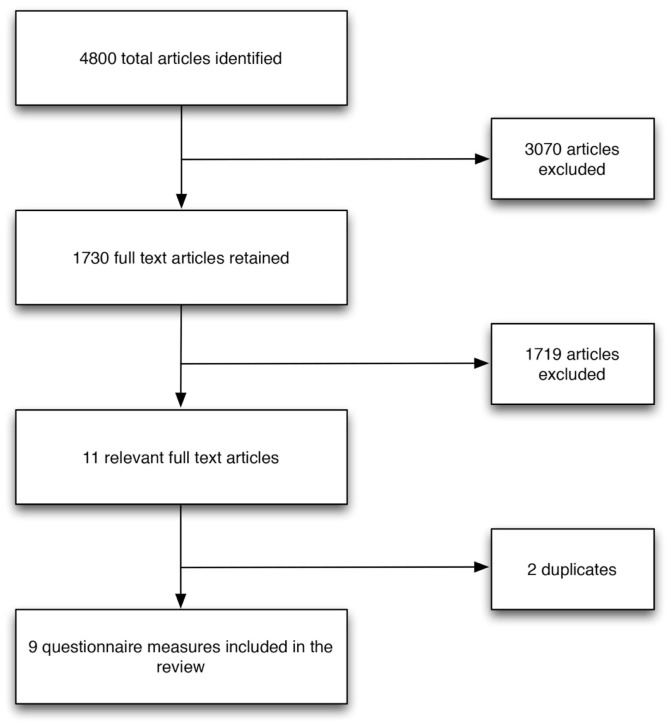
**Flow chart**.

Systematic searches yielded 4800 articles excluding duplicates, of which 11 primary research studies were identified and described nine questionnaires that measured service user and/or carer involvement in mental health care planning (Figure 1).

### Measures

The following measures were included in this review:

### COMRADE

The COMRADE measure was designed to evaluate the effectiveness of risk communication and treatment decision making in consultations (21). The tool has 20 items split across two dimensions – “Satisfaction with communication” (10-items) and “Confidence in decision” (10-items). Both the “Communication” and “Confidence” subscales are scored out of 100. The questionnaire is self-administered and was developed for general practice populations.

### Quality in psychiatric care

The Quality in Psychiatric Care (QPC) questionnaire is designed to measure quality of care in psychiatric settings from an in-patient perspective (22). The scale has 69 items that separately assess care expectations and care experiences across six domains of dignity (20 items), security (9 items), participation (13 items), recovery (16), and environment (11 items). The scale was developed using a sample of 116 patients from in-patient mental health wards in Sweden. The scale uses a 4-point Likert scale scored from 1 “Totally disagree” to 4 “Totally agree.”

### Shared decision making for in-patients with schizophrenia

This Shared Decision Making questionnaire measures patients’ perceived involvement in medical decision making in an in-patient setting (23). The scale measures user competence, interest in information, understanding, interest in participation, and decisional capacity. It is unclear how many items the scale has or whether it uses a polytomous (Likert style) scale. This measure was developed in Germany.

### User defined quality of care outcome measure

This 16 item 10-point Likert scale measures service-users self-defined quality of care (24). The measure was developed in Durham, UK for a mental health service user population.

### The decision making preference subscale of the autonomy preference index

This scale measures patients’ wish to participate in medical decisions (25). The scale was developed in the United States of America; it uses a polytomous (Likert style) scale with eight items. The scale has not been developed specifically for mental health populations.

### The treatment planning questionnaire

The Treatment Planning Questionnaire is a 49-item questionnaire part of which uses a Likert scale and part of which asks more open ended questions (26). The questionnaire asks about involvement in past treatment planning meetings and topics covered by these meetings.

### Clinical decision making scales

Clinical decision making scales consist of three scales, two with 7 items and one with 21 items (27). The questionnaires cover various aspects of clinical decision making from the point of view of the patient and the clinician. It is unclear what type of scale is used in these questionnaires. This measure was developed in the United Kingdom.

### Involvement in treatment planning survey

This questionnaire looks at interest, ability, and barriers to involvement (28). It is unclear how many items there are or whether this is a polytomous scale. The measure was developed in the United States of America for use in a mental health setting.

### User involvement in patient mental health care measure

This measure looks at democratic and assisted patient involvement, carer involvement, and management support (29). It is a polytomous measure with 30 items. The measure was developed in Norway for use in a mental health setting.

### Service user-derived acceptability criteria

Ten key criteria for PROM usability were identified via the service user and carer consultation (Table 1). Most of the identified scales met some of the criteria put forward, however none scored well across all domains. The QPC scale received the highest acceptability rating, although most service-users felt that its length prohibited its use in practice and/or its inclusion alongside other research questionnaires.

**Table 1:** **Summary of included questionnaire measures**.

Measure name and main author	Domains	User/carer rated	Published	Access	Designed for use in mental health	Developed via user/carer collaboration	Appropriate for use in secondary care settings	Version for both users and carers	Based on a social/recovery model	Multiple choice	Between 12 and 15 items	SUCAG Rating	EMPRO Score

Guidelines						Developed exclusively with mental health user/carer group			Considers wider aspects of experience, e.g., quality life, recovery, etc.	Does the scale have a polytomous (Likert-style) response option			Score out of 78
Performance in planning talk (23)	User competence	0	2	0	2	2	2	0	1	0	0	9	5
COMRADE (21)	Awareness of treatment options	2	2	2	0	2	1	0	2	2	0	13	22
User defined quality of care outcome measure (24)	Respect	2	2	2	2	2	2	0	1	2	0	15	5
The decision making preference subscale of the autonomy preference index (API) (25)	Patients wish to participate in medical decisions	2	2	0	0	0	1	0	0	2	2	11	21
Treatment planning questionnaire (26)	Frequency of treatment planning meetings	2	0	0	0	
Clinical decision making in routine care scale (CDRCCEDAR) (27)	Key aspects of CDM from the patient and clinician perspectives as they unfold in routine care	2	2	0	2	0	2	0	1	1	0	11	3
Quality in psychiatric care (QPC) (22)	Dignity (20 items)	2	2	2	2	0	2	0	2	2	0	11	16
Involvement in Treatment planning survey (28)	Interest in involvement	2	2	0	2	0	2	0	1	2	1	11	4
User involvement in in patient mental health service measure (29)	Democratic patient involvement	0	2	2	2	0	2	0	1	2	0	11	21

*0 = No, 1 = Unclear; 2 = Yes*.

### Methodological rigor

Table 1 provides an ordinal EMPRO “score” for all of the included questionnaires. Out of a possible 78 (which would be allocated to a questionnaire that met all of the EMPRO quality criteria) the mean score was 12 (±8.63, Range 3–22). The full quality table can be seen in Data Sheet 1 in Supplementary Material. The low score gives an indication of the quality of the psychometric reporting for the questionnaires included in this review, which was generally very poor. In total, only four of the included studies reported any measure of scale reliability or internal consistency (21, 22, 25, 29). None reported using any item-response theory or Rasch analysis in the development or validation of their questionnaire.

## Discussion

To date, explorations of service user and carer definitions of good care planning involvement have been limited, and are not yet sufficient to inform an assessment of whether these aspects of patient care are improved. It is crucial that questionnaires used to evaluate user and carer involvement in care planning not only make sense to service users but are also psychometrically robust, especially as important care and commissioning decisions may be informed by their scores.

Mental health services have been shown to differ from physical health services in several discrete ways. Distinguishing features of mental health services include but are not limited to a unique service history founded on aspects of containment and compulsion, and the entrenched stigmatization of their service users (30). Arguably therefore, more than in any other healthcare setting, it is vital that initiatives designed to increase service user involvement are properly evaluated against both service and patient-orientated outcomes. PROMs are now included in the majority of clinical trials and other research studies seeking to quantify the short and longer term effects of new complex interventions, health technologies, and service redesign.

The current review has identified nine different measures, each representing overlapping but subtly different definitions of care planning involvement. Of particular note, however, is the finding that all nine of these measures failed to meet a significant number of EMPRO criteria, thereby indicating that the reporting of their psychometric criteria has been largely insufficient. The expected quality of research articles reporting psychometric analyses has risen over recent years, with the increased use of item-response theories and the Rasch model in the development and validation of questionnaire measures (31). It is therefore noteworthy that PROM and scale development has not kept pace with the philosophical, policy, and practice developments occurring in contemporary mental health settings.

By definition PROs include any treatment or outcome evaluation obtained directly from patients through interviews, self-completed questionnaires, diaries, or other data collection tools. They provide patients’ perspectives on treatment benefit; and are often the outcomes of greatest importance to patients. Within the context of patient empowerment and involvement, the value of and need to collect PROMs assumes additional significance. This study has derived a new set of criteria, from the bottom-up, against which the acceptability of different measures and measurement scales can be appraised. Our findings reveal an interesting, though not wholly unexpected, disparity between the psychometric quality criteria proposed by the EMPRO measure and the more pragmatic criteria put forward by members of the service user advisory group. This disparity suggests a need to consider patient and other end-user opinions in formal assessment criteria of questionnaire quality, and to make these opinions known to other researchers, in order to improve end-user involvement in the administration and evaluation of these instruments. Our review highlights the one importance of measures that are both developed in collaboration with service-users and carers and subjected to exhaustive psychometric tests.

Historically investigators have used different instruments to capture PROs, and methods for developing, validating, and analyzing PRO data are diverse. Although not an explicit feature of all the questionnaires included in the current review, true PROM scales demand patient involvement in item generation if optimal content validity is to be ensured. This systematic review provides evidence that there are currently no psychometrically robust measures that are suitable for measuring user involvement in the mental health care planning process.

In the current review, only four of the nine identified scales were accessible in their validated formats. This may be reflective of their intended use (i.e., as an outcome in a single trial). Regardless of their intended use, it is crucial that developed scales are made readily available to potential users.

In order to improve the future measurement of involvement in care planning in mental health services, there is an urgent need to develop an acceptable and psychometrically robust measure in collaboration with the service users and carers whose perspectives and priorities it is intended to represent.

## Author Contributions

Chris J. Gibbons extracted relevant information from the included studies, contacted authors and drafted the manuscript. Penny E. Bee designed the search strategy, ran the update to the search and drafted the manuscript. Lauren Walker extracted information from included studies and contributed to the manuscript. Owen Price filtered search results and contributed to the manuscript. Karina Lovell assisted with data interpretation and drafted the manuscript. All authors assisted in revising the work for important intellectual content and approved of the final version to be published. All authors agree to be accountable for all aspects of the work presented.

## Conflict of Interest Statement

The authors declare that the research was conducted in the absence of any commercial or financial relationships that could be construed as a potential conflict of interest.

## Supplementary Material

The Supplementary Material for this article can be found online at http://www.frontiersin.org/Journal/10.3389/fpsyt.2014.00178/abstract
